# Drug Standardisation Again

**Published:** 1901-01

**Authors:** 


### DRUG STANDARDISATION AGAIN.

IN THE *Jour. of the Am. Med. Assn.* for November 24, 1900, appeared an able paper by Dr. J. Tracy Melvin, on Therapeutic Progress, which should set physicians to thinking. In support of his statement that "therapeutics is today far behind all other branches of medical science," the writer presents what he believes to be the principal causes of this condition of affairs. Yet, however well grounded these factors may be, it seems to us that at least one of them may be set aside as fanciful rather than real—namely, that "our best known and most used remedies are of uncertain and indefinite composition."

So far as medicinal chemicals are concerned, what adequate reason is there that a practitioner should employ remedies "of uncertain and indefinite composition?" Chemically pure drugs can be had by insisting that they be supplied. But are we justified in claiming as much for drugs and preparations of drugs obtained from the vegetable kingdom? The subject is worthy of more than passing thought. We are well aware that no two specimens of crude drug contain exactly the same percentage of active principle. In truth, different specimens of the same drug upon assay are frequently found to vary to such an extent that one may be ten, twenty, or even fifty times as rich in active principle as another. Consequently, we may expect as great diversity in the strength of corresponding preparations of such drugs, whenever scientific methods are not utilised to correct natural discrepancies.

The case is far from hopeless. Although, as Dr. Melvin fears, "it is practically impossible that any two samples of vegetable drugs can contain exactly the same amount of active medicinal principles," finished preparations of the same drugs should be and can be made of uniform chemical and medicinal value. In fact, some of our leading manufacturers actually do make their fluid extracts of powerful drugs conform to fixed and definite standards of strength, so that one minim or ten minims of fluid extract of opium, or fluid extract of nux vomica, always represents a definite amount of active principle, and may therefore always be expected to yield a definite therapeutic result. The well-known house of Parke, Davis & Co., for instance, who were the pioneers in drug standardisation, have for many years marketed a trustworthy line of assayed fluid extracts which are made to conform rigidly to fixed standards of alkaloidal strength. True, there are manufacturers of fluid extracts who are utterly indifferent to the necessity of standardising their products; the physician who in his clinical work does not court failure has only to let such preparations severely alone.

The objection has been raised that certain powerful drugs such as the heart tonics, digitalis, strophanthus and convallaria; the powerful arterial sedative, aconite; ergot; cannabis indica; squill, and others equally important, cannot be assayed by chemical processes. That is true. It is a scientific fact that the active principles of these drugs are decomposed by the reagents used in the assaying processes. Happily the method of physiological assay is now available, and for this important advancement in drug standardisation we are again indebted to the firm of manufacturing pharmacists previously referred to. Their experts have made practical use of the fact that certain of these drugs will produce characteristic physiological effects upon certain animals. For instance, good ergot blackens the comb of the cock, while an inferior specimen fails of effect. An active specimen of cannabis indica produces a very peculiar train of phenomena in dogs, such as disturbing the mechanism of coördination, and symptoms similar to those described in the text-books as likely to be caused by toxic doses in man. The value of the heart tonics is measured by means of delicate apparatus which accurately determines the effects of graduated doses upon the cardiac mechanism of frogs.

We are walking in the dark when we prescribe a preparation of unknown strength, the first dose of which may prove ineffective, or possibly toxic. We are compelled, virtually, to make a physiological test of the drug upon our patient. Gradually the dose is increased or diminished until we find that a definite amount produces the effect desired. But should the prescription be refilled with a preparation from another manufacturer, or by another apothecary, the correct dose must again be determined experimentally, as before.

The manufacturing pharmacist must do this work for us. He has facilities that are not available to the physician and the dispensing pharmacist. Let the manufacturer of drug extracts standardise his preparations by chemical assay when possible, and by physiological assay when the older method is inexpedient. Then the physician will be spared the humiliation of palpable impotence in the face of danger; there will be no occasion for needless experiment at the bedside, where so frequently prompt drug action saves lives that are too valuable to be imperiled by unscientific methods in pharmacy or therapeutics.

The amended Army Bill which has passed the House and is now being considered by the Senate, provides for the appointment of 200 surgeons, 50 of whom shall be majors, and 150 captains, who shall be commissioned for two years. This is but a short step in the right direction. The United States Army is woefully lacking in medical officers, and in course of time, the medical department may grow to be sufficiently large to properly care for the army.

---

The University of Pennsylvania exhibit, which was one of the most complete in the Paris exposition educational department, will be brought to Buffalo for the Pan-American Exposition.

